# Clinical Considerations of First Extensor Wrist Compartment (FEWC) Variants and De Quervain’s Disease: A Review Study

**DOI:** 10.7759/cureus.42124

**Published:** 2023-07-19

**Authors:** Dimitrios Kotzias, Christos Koutserimpas, Dimosthenis Chrysikos, Filippos Bekos, Panagiotis Georgakopoulos, George Tsakotos, Marios Salmas, Maria Piagkou, Theodore Troupis

**Affiliations:** 1 Department of Orthopaedics and Traumatology, 251 Hellenic Air Force General Hospital, Athens, GRC; 2 Anatomy, National and Kapodistrian University of Athens, Athens, GRC

**Keywords:** anatomical variations, surgical anatomy, wrist, tenosynovitis, first extensor, de quervain

## Abstract

The first extensor wrist compartment (FEWC) displays significant variants. This review highlights all possible variants that may be associated with the occurrence and pathophysiology of de Quervain’s tenosynovitis. A thorough search of PubMed and MEDLINE databases, following the PRISMA guidelines, was conducted from 2002 to 2022 to evaluate all FEWC variants, including the following: 1) the presence of an inter-tendinous septum, 2) the number of tendinous slips of the abductor pollicis longus (APL) and the extensor pollicis brevis (EPB) muscles, 3) their distal insertions and 4) the presence of a bony ridge within the FEWC. A total of 3878 wrists (1277 cadaveric and 1296 de Quervain patients) were included. Of the 1234 cadavers, a total of 701 (56.8%) were males and 533 (43.2%) were females. Regarding the 883 patients, 178 (20.2%) of them were males and 705 (79.8%) were females. An inter-tendinous septum was identified in 42.9% (47% of the patients’ wrists compared to 39.3% of the cadaveric wrists, p<0.0001). Cadaveric wrists presented two or more slips for the APL in a significantly higher percentage (92.5%, p < 0.0001) compared to de Quervain patients’ wrists (74.5%). Regarding the EPB muscle, de Quervain patients’ wrists had a single slip in 93% (p=0.0007) and two or more slips in 3.6%, compared to cadaveric wrists (a single slip in 87%, and two or more slips in 11%, p< 0.0001). A bony ridge over the radial styloid process was recorded in 58.9% of the cadaveric wrists compared to 17.8% of the patients’ wrists (p < 0.0001). Remarkable diversity concerning the structures within the FEWC was reported. The presence of an inter-tendinous septum dividing the FEWC and a single EPB muscle slip is more likely to be found in patients with de Quervain’s disease.

## Introduction and background

The first extensor wrist compartment (FEWC) is one of the most complex spaces of the upper extremity [1]. It was described as a single fibro-osseous canal, containing both tendons and tendinous slips of the abductor pollicis longus (APL) and the extensor pollicis brevis (EPB) muscles [1]. The APL and EPB tendons are single slips inserted distally at the base of the first metacarpal bone and of the proximal phalanx of the thumb [1]. However, several anatomical studies described variable forms of fibrous, tendinous, and osseous structures within this compartment associated with the etiology and pathophysiology of de Quervain’s tenosynovitis [2-4].

De Quervain’s tenosynovitis, a relatively common clinical entity, involves tendon entrapment within the FEWC [1,5-7]. Repetitive wrist motion may cause thickening of the tendons’ sheath and the APL and EPB tendons’ painful entrapment within their fibro-osseous groove over the radial styloid process. Upon presentation, patients complain about radial-sided wrist pain, exacerbated by thumb motion and ulnar wrist deviation [1].

Knowing the anatomy will help better understand the pathophysiology of de Quervain tenosynovitis and the surgical planning of this anatomical region. Operative management of de Quervain tenosynovitis is considered a common clinical practice, thus, awareness of the possible anatomical variations of the FEWC is of utmost importance for successful treatment, as well as for the avoidance of complications. 

The study aims to evaluate the prevalence of FEWC variants in de Quervain tenosynovitis and to raise the awareness of surgeons and physicians regarding these variations that may complicate treatment.

This article was previously posted to the ResearchSquare preprint server on May 5, 2023 entitled “The first extensor wrist compartment variants. Clinical considerations of the De Quervain’s disease”.

## Review

Methods

Search Strategy 

A meticulous online search of the PubMed and MEDLINE databases was conducted to identify articles regarding the FEWC variants. The study time frame was from January 2002 to December 2022. Two combined terms (and/or) were used for the literature search: “first extensor compartment”, “de Quervain”, “anatomical variations”, “abductor pollicis longus”, “extensor pollicis brevis”, “osseous variations”. Following the studies’ identification, individual references listed in each publication were further investigated for the ascertainment of additional cases.

Selection Criteria

The inclusion criteria included studies reporting cadaveric and prospective or retrospective clinical data involving 40 or more wrists. The exclusion criteria included the following: studies not published in the English language or did not present data about inter-tendinous septum (ITS), APL and EPB tendons, tendinous slips’ insertions, or bony ridge within the FEWC. Expert opinions, book chapters, or in-vitro investigations, as well as abstracts in scientific meetings, were excluded.

Data Extraction

Titles and abstracts of studies were retrieved using the search strategy; they were extracted independently by two different authors (DK & CK) who screened the titles and abstracts of the retrieved papers for eligibility and analyzed the full-text articles that met the eligibility criteria. Data extraction was performed as follows: year of publication, country of origin, sample size, and demographic characteristics of the sample (gender, and age). The identified studies were further divided into two group studies: 1) dissection and 2) clinical studies. All subjects from the cadaveric series lacked a known history of de Quervain’s tenosynovitis, while all clinical series’ patients were diagnosed with de Quervain’s tenosynovitis. The examined variables were as follows: 1) the existence of an ITS and the FEWC sub-compartmentalization, 2) the number of the APL and EPB tendinous slips, 3) the tendinous slips’ variable distal attachments, and 4) the extensor groove (EG) morphology on the radial styloid process which was categorized into three types - based on the presence of a bony ridge [8]. In type I, the EG was deep and subdivided into two grooves by a tiny bony ridge, in type II the EG had no ridge, and in type III, no EG was identified [8].

Data were recorded and analyzed using Microsoft Excel 2019 (Microsoft Corporation, Redmond, WA). Chi-squared tests were performed to compare incidences of each studied parameter between the two groups (de Quervain patients and cadavers). SPSS, version 27 (IBM Corp., Armonk, NY) was used for analysis, while p-values <0.05 were significant.

Results

**Figure 1:**
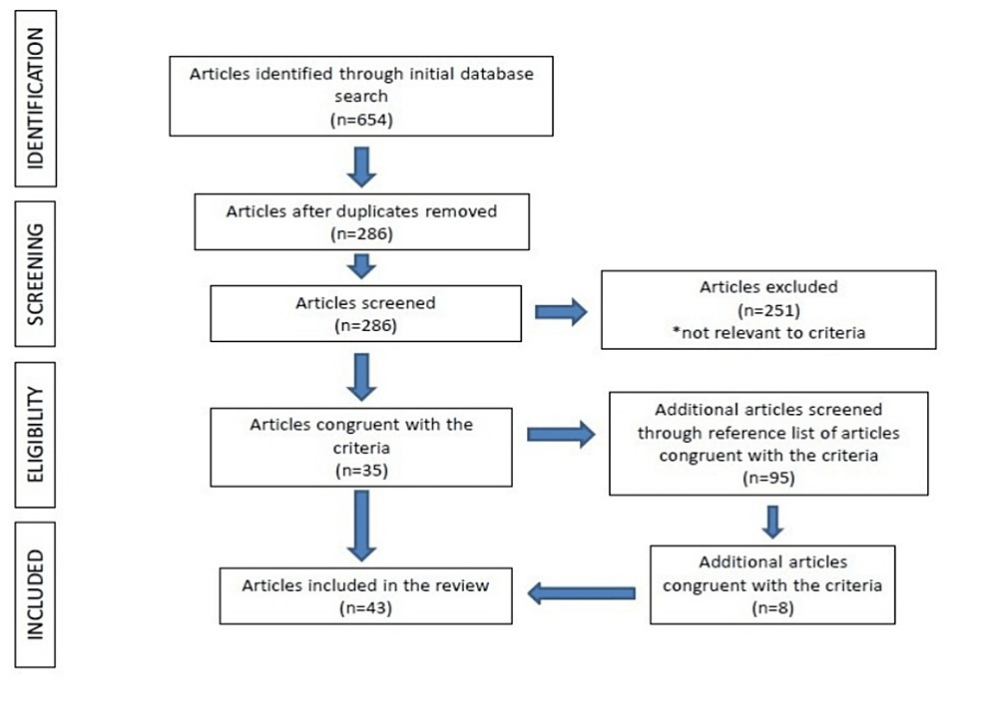
Preferred Reporting Items for Systematic Reviews and Meta-Analyses (PRISMA) search of online databases

A total of 286 articles were identified and finally, 35 of them (12.2%) met the inclusion criteria. Additionally, eight studies were retrieved after a careful investigation of the reference lists of the pertinent articles [2-44]. The search strategy is exhibited in Figure 1. In total, 3878 wrists (2564 cadaveric and 1314 from de Quervain patients) were included, as extracted from 43 studies.

The Gender in 2117 Subjects

Thirty-four out of 43 studies (79.1%) provided information about the patient' gender. From the 1234 cadavers, a total of 701 (56.8%) were males and 533 (43.2%) were females. Concerning the 883 patients, 178 (20.2%) of them were males and 705 (79.8%) were females.

Identification of the FEWC Anatomy

Regarding the clinical series, 583 out of the 1314 patients’ wrists (44.4%) were dissected, while 148 (25.4%) of these 583 wrists were evaluated by ultrasound sonography (US), before dissection. A total of 684 patients’ wrists (52.1%) had been evaluated solely by the US, and 47 (3.6%) by magnetic resonance imaging (MRI). Concerning the cadaveric studies, 2431 (94.8%) wrists were dissected, and 133 wrists (5.2%) were dissected following US imaging evaluation.

Data regarding the ITS was found in 18 cadaveric studies (1530 wrists (39.5%)) and 16 case-series (1314 wrists (33.9%)), ALP was found in 14 cadaveric studies (1012 wrists (26.1%)) and five case-series (431 wrists (11.1%)), EPB in 16 cadaveric studies (1462 wrists (37.7%)) and five case-series (430 wrists (11.1%)), and EG in four cadaveric studies (414 wrists (10.7%)) and two case-series (107 wrists (2.8%)).

The Inter-tendinous Septum in the FEWC

The presence of an ITS subdividing the FEWC into two compartments was investigated in 18 cadaveric studies (1530 wrists) and 16 case series (1314 patients’ wrists) with de Quervain patients [2-7,10-14,16-19,22-32,34,36-38,41,44]. The ITS showed a higher prevalence in patients suffering from de Quervain disease (47%, 617 out of 1314 wrists) compared to the 39.3% prevalence (602 out of 1530 cadaveric wrists; without known history of the disease) in cadavers (p-value<0.0001). Only six cadaveric studies (33.3%) and one clinical series (6.25%) characterized the ITS form as complete and incomplete [4,6,7,18,26,37,38]. The majority of these cases (76.8%, 53 out of 69 in de Quervain patients and 63.8%, 155 out of 243 cadaveric wrists; p= 0.04) had a complete ITS.

APL Tendon Anatomy

The APL tendon anatomy within the FEWC was evaluated in 14 cadaveric studies (1012 wrists) and five clinical case series with de Quervain tenosynovitis (431 patients’ wrists) [2,4,7,11,12,15,19,20,22,27,30,31,33,35,37,39,43]. All studies specified the variable number of the APL tendinous slips, except from one study [19] that did not specify the exact number of slips but reported a range (0, 1, and 2 or more tendinous slips). Cadaveric wrists had two or more tendinous slips in 936 cases (92.5%) compared to de Quervain patients’ wrists in 321 cases (74.5%, p< 0.0001).

Nine cadaveric studies reported the distal insertions of APL tendinous slips [11,15,20,31,33,35,37,39,43]. These insertions were located in the order of decreased frequency, at the first metacarpal bone base or shaft (FMBB or FMBS) in 685 (47.2%) cases, at the trapezium in 338 (23.3%), at the APB tendon in 249 (17.2%), at the opponent's pollicis muscle (OP) in 81 (5.6%), at the thenar fascia in 57 (3.9%) and the carpometacarpal joint (CMCJ) in 40 (2.8%).

EPB Tendon Anatomy

A total of 16 cadaveric studies including 1462 wrists and five case series, evaluating 430 de Quervain wrists, presented information about the number of EPB tendinous slips within the FEWC [2,4,6,7,9,11,19,21,22,26,27,30,33,34,36,37,40,41]. All studies specified the exact number of slips that varied from 0 to 4, except for one study that did not specify the exact number but reported the range (0, 1, and 2 or more tendinous slips) and one study that only reported the presence or absence of EPB [4,11]. Ten cadaveric studies and one case series examined the EPB distal attachments of tendinous slips [2,6,9,21,33,34,37,40-42].

Most de Quervain patients’ wrists (357 cases) presented a single slip (93%), 14 wrists had two or more slips (3.6%), and in 13 wrists (3%), no EPB was identified. Cadaveric wrists had a single slip in 87% (1185 cases, p= 0.0007), two or more slips in 11% (150 cases, p< 0.0001), and no EPB in 2% (27 cases, p=0.22).

Regarding de Quervain patients’ wrists, the EPB tendinous slips were attached distally at the PPB of the thumb in 87 cases (60.8%) and at the distal phalanx base (DPB) in 56 (39.2%). Concerning the cadaveric wrists, the tendinous slips were inserted distally at the proximal phalanx base (PPB) of the thumb in 496 cases (53.2%), at the extensor hood (EH) in 160 (17.2%), at the PPB and the EH in 86 (9.2%), at the DPB in 115 (12.3%), at the DPB and EH in 34 (3.7%), at the PPB and DPB in five (0.5%) each, at the PPB, EH, and DPB in 27 (2.9%) each, and at the FMBB or FMBS of the thumb in 9 (1%) per each.

EG Morphology

The variants of the radial styloid process and its EG were examined in four cadaveric studies (414 normal wrists) and two case series of de Quervain patients (107 wrists) [3,8,9,12,18,30,36]. A bony ridge over the radial styloid process was identified in a significantly higher percentage (58.9%) in 244 cadaveric wrists compared to the percentage of 17.8% in 10 patients' wrists (p< 0.0001) [3,8,12,18,30,36]. Based on Xiao et al. classification [8] the patients’ wrists were categorized as type II (46/60, 76.6%), type III (10, 16.7%), and type I (4, 6.7%). In contrast, cadaveric wrists were categorized as type I (244/414, 58.9%, p< 0.0001), type II (133, 32.1%, p<0.0001), and type III (37, 9%, p= 0.06) (Tables 1-2).

**Table 1:** Extensor groove (EG) morphology and classification of cadaveric studies based on Xiao et al. [8]

Extensor Groove Morphology in cadavers	Country	Number of cadavers	Male	Female	Mean age at death	Number of wrists	Groove with tiny bony ridge	Groove without tiny bony ridge	Type I	Type II	Type III
Rousset P et al., 2010 [36]	France	40	15	25	76	40	16	24	16	24	-
Xiao L et al., 2013 [8]	China	-	-	-	-	284	181	103	181	79	24
Gurses IA et al., 2015 [18]	Turkey	26	-	-	-	50	28	22	28	14	8
Nam YS et al., 2018 [30]	South Korea	20	10	10	80.2	40	19	21	19	16	5

**Table 2:** Extensor groove (EG) morphology and classification of clinical studies based on Xiao et al.

Extensor Groove Morphology in patients	Country	Number of patients	Male	Female	Mean age (years)	Number of wrists	Groove with tiny bony ridge	Groove without tiny bony ridge	Type I	Type II	Type III
Lee KH et al., 2014 [3]	Korea	60	-	60	45.6	60	4	56	4	46	10
Chang CY et al., 2017 [12]	USA	47	11	36	52±15	47	15	32	-	-	-

Discussion

The present review presents (in a comparative way) the incidence of all possible anatomical variants in the FEWC from both clinical studies (with known de Quervain disease) and cadaveric studies (without known medical history). All included studies included more than 40 subjects to avoid taking into account random findings of cases that may overestimate the total incidence of variants.

The present review has shown remarkable morphological variants in the FEWC. The most common variants were the presence of complete or incomplete ITS, the presence of multiple EPB and APL tendinous slips with various distal attachments, and a tiny bony ridge over the radial styloid process. These variants are considered to be the main common anatomical findings correlated with the occurrence of de Quervain’s tenosynovitis [3,4]. Repetitive wrist motion and acute trauma are associated with myxoid degeneration and fibrous tissue deposits, causing thickening of the tendons’ sheath and painfully entrapping of the APL and EPB tendons within their fibro-osseous groove over the radial styloid process [1]. The presence of certain variants, such as the ITS, the multiple EPB, and APL tendinous slips with their variable distal attachments and the existence of a bony ridge over the radial styloid process, may compress the narrow FEWC and alter its biomechanics, and consequently reproduce high amounts of friction [1,3,4,8,12,37].

In the current study, the most common variant of FEWC was the presence of an ITS, which subdivided the FEWC into two compartments. The septum had significantly higher prevalence in patients suffering from de Quervain disease (47%) compared to the cadaveric wrists (39.3%). Consequently, it seems that this fibrous septum may be a significant factor contributing to the pathophysiology of de Quervain’s. The FEWC sub-compartment usually contains the EPB tendon, located at its dorsal, ulnar aspect or less commonly within its osseous floor [2,10,37]. The EPB tendon sub-compartment is reported to be smaller than the adjacent APL tendon sub-compartment, and it may be more prone to friction since the EBP tendon is more mobile during thumb movement [18,25]. Furthermore, the ITS may be incomplete with no extension along the entire FEWC length. When the septum is incomplete, it is mainly located distally and may be less evident during imaging or surgical exploration [4,7,13,18,21,24]. Both patients suffering from de Quervain tenosynovitis and cadavers had higher incidence of complete ITS. However, complete ITS was observed in significantly higher percentage of patients with de Quervain disease. Thus, a high index of suspicion of this variant and meticulous inspection of the FEWC is of paramount importance for the successful surgical treatment of the disease. The presence of an ITS may also represent a factor of conservative treatment failure [17,25]. The treatment with a steroid injection into the FEWC may often have unsatisfactory results up to 38% [45,46]. Due to the ITS existence, the injection may infiltrate the more superficial compartment and not the usually smaller and deeper one containing the EPB tendon [45-47].

Among the tendons traversing the FEWC, the APL displays the greatest diversity, considering the number of tendinous slips and their distal insertions. The APL was reported to display 1 to 7 distal tendinous slips. The accessory slips were usually located radial to the main slip. The incidence of two or more slips was significantly lower in patients with de Quervain disease when compared to cadavers. Therefore, it seems that the incidence of multiple APL tendinous slips may be the rule rather than the exception [4,31,36,37].

Multiple EPB tendinous slips variants and their distal attachments have also been evaluated. The present review has shown that an EPB with a single tendinous slip is significantly more prevalent in de Quervain patients, compared to cadavers without known history of the disease. Furthermore, the EPB was absent more frequently in in patients’ wrists but not to a significant degree. Furthermore, the EPB muscle belly may be completely distinct or fuse to a variable extent with the APL muscle belly. Consequently, the presence of such a fusion may suggest that phylogenetically EPB could differentiate with APL from a common muscle [4,6,20,33,48].

Furthermore, in the current study, the incidence of a bony ridge over the radial styloid was found to be significantly lower in patients with de Quervain, compared to cadavers. The incidence of a bony ridge dividing the EG is associated with the presence of an ITS [3,8,9,18,36]. Hence, it could attribute to the pathophysiology of the disease. Nevertheless, it is unclear whether this plays an important role and further research is needed.

The present study has some limitations. The heterogeneity of the included studies and the inability to directly correlate results of the cadaveric series with clinical findings represents the main limitation. Cadavers had no known history of de Quervain tenosynovitis, therefore, we decided to use this sample as a control group and present the data in a comparative way between the population with known de Quervain disease (clinical studies) and the population without any available medical history (cadaveric studies). Moreover, different techniques were used for the identification of the anatomical variants including surgical dissection and imaging techniques. Finally, some studies did not define the exact number of tendinous slips or the EG type. Nevertheless, the present review included a significant number of both cadaveric and de Quervain wrists. Data regarding all variants and a comparison of the prevalence between two groups (de Quervain patients and cadavers) were provided, leading to meaningful observations regarding the pathophysiology of the disease as well as some clinical implications during treatment. Hence, the study provides a thorough insight into the FEWC surgical anatomy.

## Conclusions

The review study summarizes all variants of the FEWC and associates them with the etiology and management of de Quervain’s tenosynovitis. Significant diversity exists concerning the ITS presence, the number of APL and EPB tendinous slips, and their distal attachments, as well as the EG extensor morphology. A thorough knowledge of the possible variants and the anatomy of the FEWC is of paramount importance since it will help better understand the pathophysiology of de Quervain tenosynovitis and surgical planning of this anatomical region. Awareness of the possible anatomical variations of the FEWC is of utmost importance for successful treatment, as well as for the avoidance of complications.
